# Cardiac hypoxic resistance and decreasing lactate during maximum apnea in elite breath hold divers

**DOI:** 10.1038/s41598-021-81797-1

**Published:** 2021-01-28

**Authors:** Thomas Kjeld, Jakob Møller, Kristian Fogh, Egon Godthaab Hansen, Henrik Christian Arendrup, Anders Brenøe Isbrand, Bo Zerahn, Jens Højberg, Ellen Ostenfeld, Henrik Thomsen, Lars Christian Gormsen, Marcus Carlsson

**Affiliations:** 1 Department of Radiology, Herlev Hospital, University of Copenhagen, Herlev Ringvej 75, 2730 Herlev, Denmark; 2 Department of Anesthesiology, Herlev Hospital, University of Copenhagen, Copenhagen, Denmark; 3 Department of Cardiothoracic Surgery, Rigshospitalet, University of Copenhagen, Copenhagen, Denmark; 4 Department of Clinical Physiology and Nuclear Medicine, Herlev Hospital, University of Copenhagen, Copenhagen, Denmark; 5 Department of Cardiothoracic Anesthesiology, Rigshospitalet, University of Copenhagen, Copenhagen, Denmark; 6 Department of Clinical Sciences Lund, Lund University, Skåne University Hospital, Lund, Sweden; 7 Department of Nuclear Medicine & PET Centre, Aarhus University Hospital, Aarhus, Denmark

**Keywords:** Cardiovascular biology, Cardiovascular biology

## Abstract

Breath-hold divers (BHD) enduring apnea for more than 4 min are characterized by resistance to release of reactive oxygen species, reduced sensitivity to hypoxia, and low mitochondrial oxygen consumption in their skeletal muscles similar to northern elephant seals. The muscles and myocardium of harbor seals also exhibit metabolic adaptations including increased cardiac lactate-dehydrogenase-activity, exceeding their hypoxic limit. We hypothesized that the myocardium of BHD possesses similar adaptive mechanisms. During maximum apnea ^15^O-H_2_O-PET/CT (n = 6) revealed no myocardial perfusion deficits but increased myocardial blood flow (MBF). Cardiac MRI determined blood oxygen level dependence oxygenation (n = 8) after 4 min of apnea was unaltered compared to rest, whereas cine-MRI demonstrated increased left ventricular wall thickness (LVWT). Arterial blood gases were collected after warm-up and maximum apnea in a pool. At the end of the maximum pool apnea (5 min), arterial saturation decreased to 52%, and lactate decreased 20%. Our findings contrast with previous MR studies of BHD, that reported elevated cardiac troponins and decreased myocardial perfusion after 4 min of apnea. In conclusion, we demonstrated for the first time with ^15^O-H_2_O-PET/CT and MRI in elite BHD during maximum apnea, that MBF and LVWT increases while lactate decreases, indicating anaerobic/fat-based cardiac-metabolism similar to diving mammals.

## Introduction

The physiological adaptions in breath hold divers (BHD) to apnea, includes significant diving responses, attenuated post-apnea acidosis, reduced sensitivity to oxidative stress and, probably critical to their performances, reduced sensitivity to hypoxia^1–6^. It has also been demonstrated that similar to adaptions in the northern elephant seal, which can endure apnea for more than 120 min, BHD have a non-coupled maximum respiratory state in their skeletal muscles ~ 22–23% lower than matched aerobic athletes^7^. Hence, the skeletal muscles of BHD possess an oxygen conserving adaptation, and this might—at least partly—explain, the extreme performances of BHD holding their breath for more than 11 min and swimming as far as 300 m, or diving beyond 200 m in depth, all on a single breath of air (http://www.freedive-earth.com/aida-freediving-world-records). Similarly, samples of the skeletal muscles and the heart from adult harbor seals (Phoca Vitulina) shows up to sixfold higher β-hydroxyacyl-CoA dehydrogenase activities normalized to tissue-specific resting metabolic rate, and the heart of the seals possess the highest lactate dehydrogenase activity, compared to dog and rat, suggesting both a reliance on anaerobic metabolism during dives exceeding the animal's aerobic limit, but also a high aerobic capacity for lipid metabolism within the heart^8^. It could be hypothesized, that the hearts of BHD would possess similar adaptions. However, two cardiac magnetic resonance imaging (CMR) studies of BHD reported that after only 4 min of dry apnea concomitant with a reduction in arterial oxygen saturation ~ 23%, cardiac troponins increase, and CMR epicardial perfusion decreases about 60%^9,10^. We have reported of elite BHD enduring a reduced arterial oxygen saturation of more than ~ 33%^4^, and after competitions with pool static apneas of ~ 5 min (and subsequent 88 ± 21 m underwater swim), where cardiac troponins remains unchanged normal^11^. The explanation might be, that BHD who can endure apnea for more than 4 min have a lesser increase in ischemia-modified albumin (IMA), a marker of the release of reactive oxygen species (ROS) and a measure of resistance to hypoxemia, than compared to BHD who can endure less than 4 min of apnea^12^. This is in accordance with studies of adult seals are demonstrated to have a higher relative capacity for mitochondrial respiration than pups and juvenile seals^13^, and adult diving mammals also have resistance to release of ROS during dives to tolerate ischemia^14,15^, indicating that the hypoxic capacity is an adaption.

Hence, it is hypothesized that similar adaptive mechanisms to hypoxia as observed in the hearts of adult diving mammals could be observed in the hearts of human elite BHD, who can endure apnea for more than 4 min. This study aimed to demonstrate this hypothesized adaptation in BHD. As myocardial ischemia is dependent on both blood oxygen levels, perfusion and the need of oxygen based on cardiac contraction, these parameters were quantified using arterial samples, cine CMR and ^15^O-H_2_O-PET/CT and the resulting myocardial oxygenation as a sign of ischemia measured using BOLD CMR at rest and during a maximal voluntary apnea in elite BHD with a self-reported maximum static apnea of more than 5 min.

## Methods

This study included eight Danish healthy male non-smoking subjects and was approved by the Regional Ethics Committee of Copenhagen (H-1-2013-060). All clinical investigations were conducted according to the principles expressed in the Declaration of Helsinki. Informed consent, written and oral, were obtained from the participants.

All subjects were elite BHD (age 42 ± 8 years) able to hold their breath for more than 5 min and ranked among the national top 10. Three ranked among the World top 10, one was a 2016 outdoor free-diving World champion, one reached third place at the same championship (no limit depth competition), and one was a World record holder.

### VO_2_ max measurement and dual-energy X-ray absorptiometry scan

Subjects completed a standardized warm-up followed by an incremental cycling test starting at a workload of 150 W and increasing 25 W every minute until voluntary exhaustion. Pulmonary O_2_ and CO_2_ concentrations in the expired gas were continuously measured breath-by-breath (Quark, Cosmed, Rome, Italy) during the test. The highest recorded 30 s average oxygen uptake (VO_2_) during the test was defined as VO_2_max. For recognition of true VO_2_max three of five criteria had to be met: individual perception of exhaustion, respiratory exchange ratio > 1.15, plateau of VO_2_ curve, heart rate approaching age-predicted maximum and inability to maintain a pedaling frequency above 70 rpm. A dual-energy X-ray absorptiometry scan (Lunar iDXA; Lunar, Madison, WI, USA) was performed to assess body composition.

### Cardiac magnetic resonance imaging (CMR)

#### Image acquisition

For one day prior to the study, the subjects refrained from physical exercise and consumption of alcohol or caffeine. Imaging was performed in a 1.5 T MR imaging system (Achieva, Philips Medical System, The Netherlands) after a warm-up of three consecutive apnea to maximize the diving response^4^. Cine images were acquired at (1) rest during a short (< 20 s) apnea at end-expiration with open pharynx, (2) end of a apnea with max expiration and (3) the end of dry static apnea after glossopharyngeal insufflation (GPI)^16^. Images were collected shortly before end of apnea before breathing, and subjects were instructed to stay as calm as possible during imaging to avoid imaging artefacts. Cardiac chamber volumes and function were collected in the transversal and double-oblique short axis stacks with 8 mm thick slices and 25% gap. Cine imaging were performed with retrospectively ECG-gated steady-state free precession sequences (SSFP) reconstructed to 25 phases covering the entire cardiac cycle using the following settings: TR/TE 3.3/1.6 ms, flip angle 60°; and spatial resolution 1.3 × 1.3 × 8 mm^3^. For blood oxygen level dependence (BOLD) imaging a fast gradient-echo multi-echo black blood T2* sequence was used in the short axis plane^17,18^. The BOLD sequences were acquired at rest and after 240 s of apnea, to ensure image collection on all subjects, as the duration of this could be up to 20 s. BOLD had the following settings: TR 25.8 ms with effective repetition time 1600 ms and collecting 14 images with TE from 3.2 to 24.1 ms, flip angle 20°; and spatial resolution 1 × 2 × 1.2 × 8 mm^3^.

#### Image analysis

Left and right ventricular (LV and RV) and atrial (LA and RA) volumes were determined in end-diastole (ED) and end-systole (ES) and used to determine the influence of apnea on cardiac volumes, function, filling and individual chamber outflow. Data were analyzed by one experienced level three CMR reader and one level one CMR cardiologist using dedicated software (Segment version 2.1) carefully avoiding image artefacts and imaging especially during involuntary breathing movements^19^. LV wall thickness (LVWT) was determined from a midventricular short-axis slice from the epicardial and endocardial borders delineations of the left ventricle at end-diastole. Stroke volume (SV) was computed as the difference between end diastolic volume (EDV) and end systolic volume (ESV). Ejection fractions were computed as SV divided by EDV. T2* was determined from the BOLD images using a LV septal midwall region of interest carefully avoiding image artefacts and using a fitting algorithm in the Segment software as previously described^20^.

### Arterial blood gases and hemodynamic data

On a separate day, after a warm-up of three consecutive apneas to maximize the diving response^4^, all subjects performed a maximum static pool apnea to simulate apnea during natural circumstances; (1) before, (2) after 4 min of apnea, and (3) just before end of maximum apnea, arterial blood samples were obtained through a catheter (1.1 mm, 20 gauge) in the radial artery of the non-dominant arm with connection to a transducer for continuous flow of saline (Baxter, Uden, the Netherlands). Blood gas analyses were performed immediately after sampling, using an automated self-calibrating blood gas machine (ABL 725, Radiometer, Copenhagen, Denmark) and evaluated for pH, bicarbonate, lactate, PaCO_2_, and the arterial oxygen tension (PaO_2_).

Arterial blood pressures and heart rates were recorded concomitantly using a Lifepack ® 20 monitor.

### Lipids

Blood samples for lipid analysis were collected following 8 h of fasting from the radial artery as described above for arterial blood gas collection, and before the pool dive. Total Cholesterol, High-density lipoprotein, Low-density lipoprotein and Triglycerides were measured with an automated analyzer (Roche Integra 400, Roche Diagnostics, Switzerland) using commercially available kits.

### ^15^O-H_2_O-PET/CT perfusion determined myocardial metabolism

#### Imaging protocol and image reconstruction

The six subjects with the longest pool maximum apneas, were recruited for an additional ^15^O-H_2_O-PET/CT study. The participants in the ^15^O-H_2_O-PET/CT sub-study were required to be able to hold their breath for 5 min while lying in the PET/CT scanner in the supine position with arms raised above the head. They were instructed to refrain from intake of chocolate, to refrain from strenuous physical exercise for one day and to be fasting for at least 6 h before the study.

**Figure 1:**
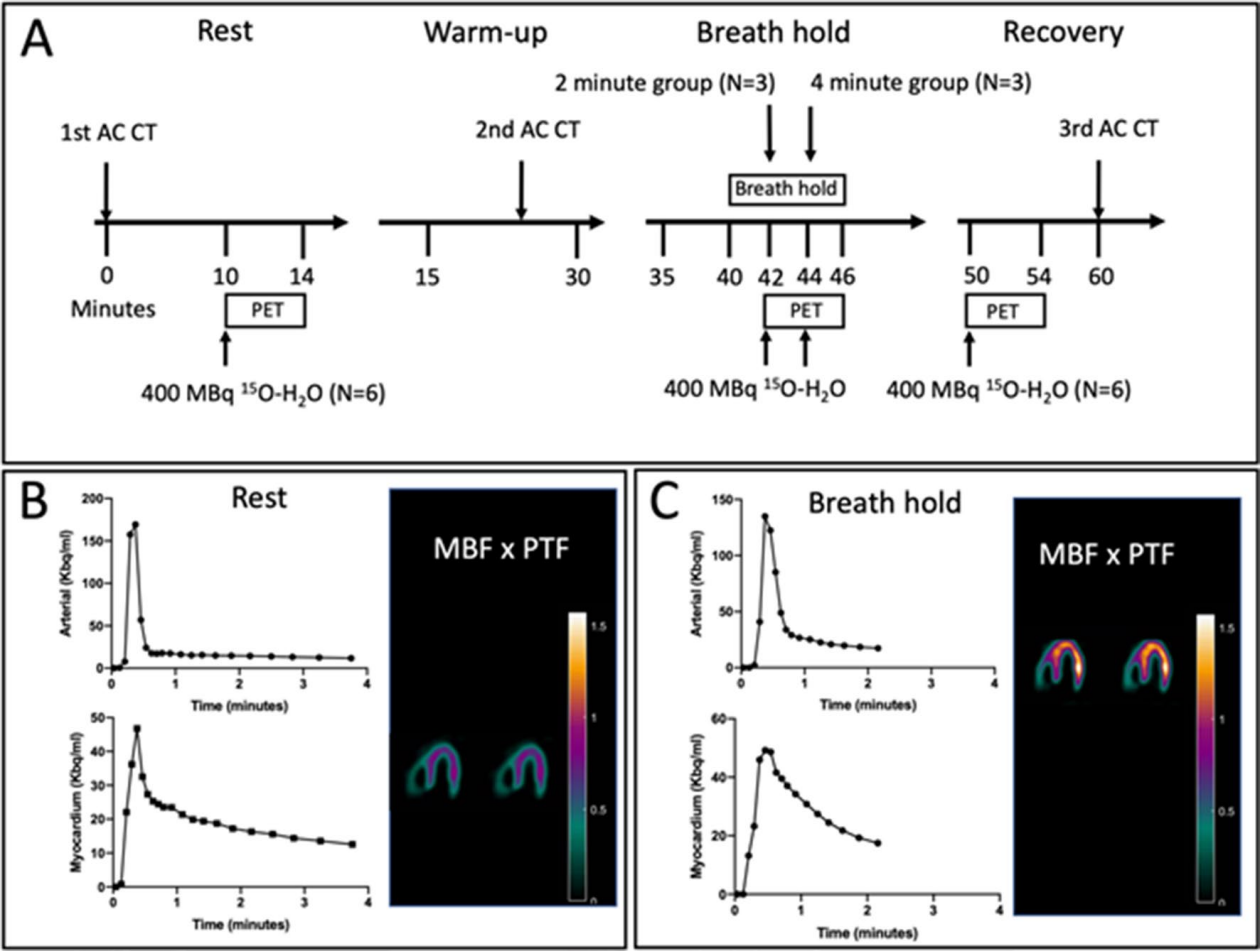
(**A**) ^15^O-H_2_O-PET/CT study day flowchart. The resting and recovery perfusion ^15^O-H_2_O-PET/CT scans were performed in all participants, whereas the apnea perfusion ^15^O-H_2_O-PET/CT was performed with participants divided into two groups with radiotracer bolus injected at 2- and 4-min after initiation of apnea. The average breath hold of all participants were 378 ± 77 s. (**B**) Example of input function (arterial radioactivity, upper row) and myocardial time-activity curves (lower row) in a representative participant. As seen, the entire time-activity curve of the participant has been captured allowing for precise calculation of MBF and PTF. In the right column, horizontal long axis slices with calculation of MBF × PTF allows for parametric visualization of radiotracer uptake in the left ventricle. (**C**) Input function and myocardial time-activity curve for the same subject during apnea. Due to cessation of apnea approximately 2-min after injection of radiotracer (the participant was in the 4-min group), the entire myocardial time-activity curve was not recorded. However, the inflow of tracer was accurately recorded allowing for accurate determination of MBF × PTF, evident from the correct delineation of the left ventricle on the horizontal long axis slices (right side column).

^15^O-H_2_O-PET/CT data were obtained in list mode on a GE Discovery MI Digital Ready PET/CT system (GE, Milwaukee, WI, USA). The participants underwent three image acquisitions: (1) at rest, (2) during hyperemia induced by a dry static apnea after GPI^16^ and after a warm-up of three consecutive apneas to maximize the diving response^4^, and (3) third in the recovery phase 4 min after the apnea (Fig. 1). For each acquisition, 400 MBq ^15^H_2_O was administered intravenously in an antecubital vein as a single bolus using an automated injection system (Medrad Stellant, Bayer, Leverkusen, Germany). For the stress acquisition, ^15^H_2_O was infused after 2 min (n = 3) or 4 min (n = 3) after initiation of the apnea with acquisition of data for at least an additional 2 min (Fig. 1). Average apnea duration was 378 ± 77 s. For each PET scan, an attenuation CT was performed at time points allowing for correct co-registration of the PET and CT images. The resting and recovery attenuation correction CT’s were thus performed immediately prior to and after the PET scans, whereas the apnea attenuation correction CT was performed during warm-up mimicking maximal apnea.

#### Image analysis

Myocardial blood flow (MBF) was quantified by ^15^O-H_2_O-PET/CT on a GE Discovery MI Digital Ready PET/CT system (GE, Milwaukee, WI, USA) at time points as specified above. The 4-min dynamic images were reconstructed in a 3.27 × 3.27 × 3.27 mm matrix utilizing all normal corrections (attenuation, scatter, dead time and randoms) and the VPFX-S reconstruction algorithm. For subsequent analysis, the dynamic scan was divided into 21 frames (1 × 10, 8 × 5, 4 × 10, 2 × 15, 3 × 20 and 2 × 30 s). Kinetic analyses were performed using aQuant software (MedTrace Pharma, Lyngby, Denmark) and a validated 1-tissue compartment model with image derived input from cluster analysis, corrections for spillover and automatic estimation of MBF and perfusable tissue fraction (PTF)^21^. In healthy subjects, PTF primarily reflects the incomplete estimation of true tissue radiotracer uptake (the partial volume effect), which is a particular challenge in subjects with diaphragmatic movements and therefore reported. This parameter is derived from the tracer inflow, whereas MBF is derived from the outflow curve (see Fig. 1B for examples).

### Statistical analysis

To test for imbalances in the variables in our study with a limited number of subjects, we performed a power calculation: A P-value < 0.05 was considered statistically significant. Given this level of significance, our sample size of 6 subjects in the ^15^O-H_2_O-PET/CT sub study resulted in a power of 80% to detect an expected increase in MBF of at least 1 ml/g/min with a standard deviation of 0.4^22^.

Variables are presented as mean ± standard error of the mean (SEM). Data were analyzed by Sigma-Plot ® using one-way repeated measures ANOVA. Kruskal–Wallis with Tukey’s methods posthoc respectively were used to evaluate differences between the collected data during rest, apnea, and recovery. Time-related changes in MBF, PTF and MBF × PTF interaction in the 2- and 4-min groups were analyzed using a mixed model repeated measures ANOVA.

## Results

### VO_2_ max and dual-energy X-ray absorptiometry scan

The morphometric variables age, height, body mass, whole-body aerobic capacity (VO_2_max) and lipids, are displayed in Table 1.

**Table 1:** Subject characteristics.

Parameter	Result
No. subjects	8 male BHD
Static personal best (seconds)	395 ± 48
Dynamic pool personal best (meters)	171 ± 38
Dynamic pool no fins personal best (meters)	143 ± 38
Age (years)	43 ± 8
Height (cm)	186 ± 8
Weight (kg)	80 ± 6
Body surface area	2.0 ± 0.1
Body Mass Index	23.1 ± 1.9
Fat mass %	19.0 ± 3.4
Bone mineral content (kg)	3.2 ± 0.3
Fat (kg)	15.2 ± 3.7
Fat free mass (kg)	62.1 ± 4.2
Bone mineral density (kg/m^2^)	1.3 ± 0.1
T/Z-score (+ 1 to − 1)	0.8 ± 1.0
Maximal oxygen uptake (ml O_2_/min)	3672 ± 672
Capillary Hemoglobin (g/dl)	15.2 ± 0.8
Venous Hemoglobin (calculated^66^, g/dl)	14.6 ± 0.7
Cholesterol mmol/l	4.8 ± 0.9
HDL mmol/l	1.3 ± 0.4
LDL mmol/l	3.1 ± 1.2
Triglycerid	1.1 ± 0.3
Creatinine mmol/l	81 ± 14

Basic morphometric data. Values are mean ± Standard Error of Means.

*BHD* breath hold divers, *HDL* High density lipoprotein, *LDL* low density lipoprotein.

### Pool apnea

After GPI and 326 ± 19 s of static apnea in a pool, heart rate (HR) decreased from 86 ± 5 bpm to 50 ± 13 beats/min (P < 0.001, Tables 2, 3), while Mean Arterial Blood pressure (MAP) increased from 103 ± 4 mmHg to 148 ± 15 mmHg (P < 0.001), and PaCO_2_ rose from 5.2 ± 0.2 kPa to 7.0 ± 0.2 kPa (P < 0.001) concomitant with a decrease in PaO_2_ from 12.2 ± 0.5 kPa to 4.3 ± 0.4 kPa (P < 0.001), and a decrease in pH from 7.42 ± 0.029 to 7.35 ± 0.01 (P < 0.001) and a decrease in lactate from 1.70 ± 0.13 to 1.36 ± 0.18 (P = 0.013).

**Table 2:** Hemodynamic parameters and blood gas results at rest, after 4 min apnea and at end of apnea (326 ± 19 s).

	Rest	4 min apnea	Max apnea	After apnea
MAP/mmHg	81 ± 5	87 ± 7	58 ± 4 *	P < 0.001
HR/beats min^−1^	117 ± 4	113 ± 10	60 ± 3 *	P < 0.001
SaO_2_/ %	7.4 ± 0.4	7.3 ± 0.6	4.9 ± 0.4 *	P < 0.001
Neck saturation/%	59 ± 1	56 ± 1	51 ± 2 *	P = 0.003
pH	207 ± 8	202 ± 16	128 ± 7 *	P < 0.001
PaCO_2_/kPa	93 ± 6	93 ± 6	73 ± 5 *	P < 0.001
PaO_2_/kPa	114 ± 4	109 ± 10	56 ± 3 *	P < 0.001
SBE	55 ± 1	53 ± 1	44 ± 1 *	P < 0.001
Bicarbonate	35 ± 3	37 ± 8	24 ± 2 *	P = 0.013
Lactate	79 ± 5	102 ± 6 *	46 ± 5 *	P < 0.042

Values are means ± Standard Error of Means. *P < 0.05 vs. rest and ***P < 0.001.

*MAP* mean arterial pressure, *HR* heart rate, *SBE* standard base excess, *SaO*_*2*_ arterial oxygen saturation. *PaCO*_*2*_ partial pressure of carbondioxide, *PaO*_*2*_ Partial pressure of oxygen.

### CMR

**Figure 2:**
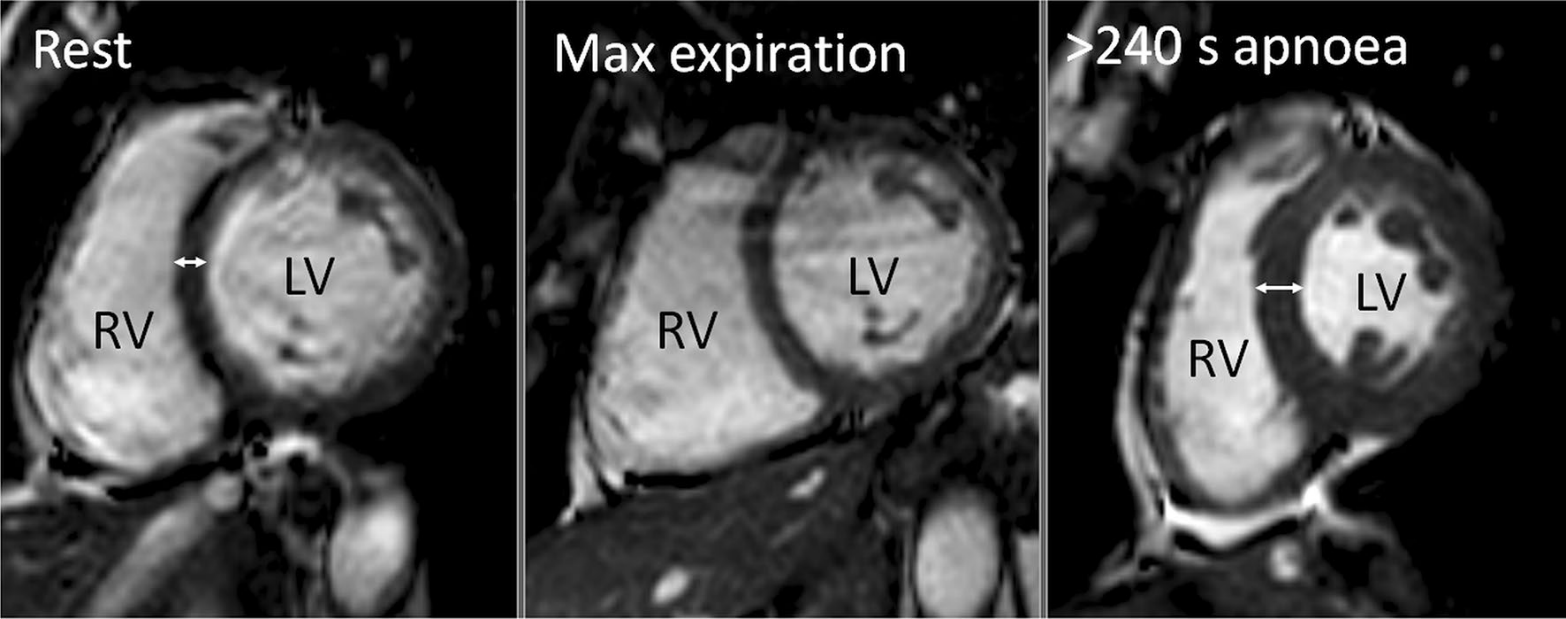
Example of Cardiac MR imaging of one subject, displaying a midventricular short axis view at papillary muscle level at end-diastole. Compared to rest (left panel), the left ventricle (LV) and right ventricle (RV) are similar in size at end of apnea with maximum expiration (center panel). In contrast, at the end of > 240 s of apnea with glossopharyngeal insufflation (right panel) there is a decrease in LV and RV size and thickening of the ventricular wall (double-headed arrows) compared to rest.

CMR after 240 s of apnea with GPI, revealed that ventricular volumes decreased, both right (~ 38%, P < 0.001, Table 3) and left (~ 40%, P = 0.001). Atrial volumes decreased as well, both right (~ 48%, P < 0.01) and left (~ 31%, P < 0.05), Right and left stroke volumes decreased (right ~ 51%, P < 0.001, left ~ 49%, P < 0.001) as did left ventricular cardiac output (~ 34%, P = 0.001). At the end of 240 s of apnea with GPI a decrease was observed in right and left ventricular ejection fraction (RVEF rest 53 ± 1% vs. BH 44 ± 1%, P < 0.001 and LVEF rest 59 ± 1% vs. apnea 51 ± 2%, P = 0.003), concomitant with an increase in LV wall thickness (~ 31%, P < 0.005, Fig. 2).

**Table 3:** Cardiovascular variables and organ volumes as evaluated with Cardiac Magnetic Resonance imaging at rest, at end of apnea with maximal expiration and at end of 240 s of apnea with glossopharyngeal insufflation (GPI) in 8 elite breath-hold divers.

Parameter	Rest	End of apnea with max expiration	End of apnea with GPI	P-value: GPI compared to rest
LV EDV, ml	198 ± 8	199 ± 16	119 ± 6 *	P < 0.001
LV ESV, ml	81 ± 5	87 ± 7	58 ± 4 *	P < 0.001
LV SV, ml	117 ± 4	113 ± 10	60 ± 3 *	P < 0.001
LV CO, l/min	7.4 ± 0.4	7.3 ± 0.6	4.9 ± 0.4 *	P < 0.001
LV EF, %	59 ± 1	56 ± 1	51 ± 2 *	P = 0.003
RV EDV, ml	207 ± 8	202 ± 16	128 ± 7 *	P < 0.001
RV ESV, ml	93 ± 6	93 ± 6	73 ± 5 *	P < 0.001
RV SV, ml	114 ± 4	109 ± 10	56 ± 3 *	P < 0.001
RV EF, %	55 ± 1	53 ± 1	44 ± 1 *	P < 0.001
LA EDV, ml	35 ± 3	37 ± 8	24 ± 2 *	P = 0.013
LA ESV, ml	79 ± 5	102 ± 6 *	46 ± 5 *	P < 0.042
RA EDV, ml	56 ± 9	59 ± 10	29 ± 4 *	P = 0.009
RA ESV, ml	104 ± 12	131 ± 10	40 ± 4 *	P < 0.001
BOLD, ms	33.7 ± 2.8	NA	32.6 ± 1.9	P = 0.453
LVWT, mm	7.5 ± 0.44	NA	9.8 ± 0.51	P < 0.005

Values are means ± Standard Error of Means. *P < 0.05 vs. rest.

*GPI* glossopharyngeal, insufflation; *LV* left ventricular; *RV* right ventricular; *EDV* end-diastolic volume; *ESV* end-systolic volume; *SV* stroke volume; *CO* cardiac output; *EF* ejection fraction; *LA* left atrial volume; *RA* right atrial volume; *BOLD* blood oxygen level dependent, *LVWT* left ventricular wall thickness.

T2* BOLD did not change.

Results at end of apnea with max expiration did not change compared to rest, except for LA ESV (Table 3).

### Myocardial perfusion imaging (^15^O-H_2_O-PET/CT)

MBF increased after 4 min of apnea from 0.97 ± 0.15 ml/min/g at rest to 2.15 ± 0.39 ml/min/g during apnea (378 ± 77 s) induced stress (P = 0.001; Table 4), whereas PTF decreased from 0.67 ± 0.09 at rest to 0.58 ± 0.1 during apnea (P = 0.03, Table 4).

**Table 4:** Myocardial blood flow (MBF) as evaluated with ^15^O-H_2_O-PET/CT at rest, during apnea of 378 ± 77 s and at recovery in 6 elite breath-hold divers.

	Rest (n = 5)	2 min apnea (n = 3)	4 min apnea (n = 3)	Recovery (n = 5)	P-value *
MBF (ml/g/min)	0.97 ± 0.15	0.82 ± 0.24	2.15 ± 0.39	1.02 ± 0.20	0.002
PTF	0.67 ± 0.09	0.71 ± 0.09	0.58 ± 0.10	0.68 ± 0.09	0.03
MBF × PTF (ml/g/min)	0.65 ± 0.17	0.58 ± 0.13	1.24 ± 0.23	0.70 ± 0.18	0.001

Values are means ± Standard deviations. *Mixed model repeated measures ANOVA. Rest compared to apnea.

*MBF* myocardial blood flow, *PTF* perfusable tissue fraction.

## Discussion

The main and novel findings of our study are: After a warm-up of three consecutive apneas and 1) during 4 min of apnea, the hearts of BHD in this study were without signs of concomitant T2* BOLD assessed myocardial ischemia on CMR, 2) after 4 min of apnea ^15^O-H_2_O-PET/CT revealed increased myocardial blood flow to levels approaching theoretical maximum hyperemia^23–26^ without signs of regional myocardial perfusion deficits, and 3) at end of max pool apnea, systemic oxygenation decreased to 4.3 kPa ± 0.4 and pH decreases concomitant with a decrease in lactate in BHD. This has to our knowledge not previously been described. Hence, we demonstrated for the first time that BHD, similar to adult diving mammals, possess cardiac hypoxic resistance and increased myocardial blood flow with CMR and ^15^O-H_2_O-PET/CT during apnea ~ 6 min, and also lactate metabolization during max pool apnea. Our study also demonstrated that at end of max apnea with GPI, mean arterial blood pressure increased, and HR, biventricular EDV, CO and EF decreased, concomitant with an increase in LV wall diameter and an increase in PaCO_2_.

Guensch et al. demonstrated, that when PaCO_2_ is increased, myocardial perfusion and oxygenation remains preserved or even increased during apnea episodes of 60 s in anaesthetised animals and during voluntary apnea of up to 117 s in healthy humans^27,28^: Longer apneas causes higher increases in BOLD-signal intensity and a decline in capillary PO_2_ is compensated through a CO_2_-mediated increase in MBF as a countermeasure for desaturation and prevention of compromised myocardial oxygenation during long effective apneas^28^. During repetitive apnea in BHD combined with 100 W exercise, it has been demonstrated that PaO_2_ decreases to 7.3 kPa and PaCO_2_ increases to 8.2 kPa^4^ concomitant with a decrease in pH to 7.35. In this study, PaO_2_ decreased to 4.3 ± 0.4 kPa while PaCO_2_ increased to 7.0 ± 0.2 kPa and pH decreased to 7.35 during max apnea (326 ± 19 s). In part, this adaption to hypoxia and hypercapnia may explain the findings of sustained myocardial oxygenation and the increased MBF during apnea in our study, and the latter is comparable to increases in MBF during max exercise in endurance trained athletes^29^.

Kyhl et al. reported that myocardial perfusion index decreased 60% after ~ 4 min of apnea^10^, and Eichhorn et al. reported that 4–5 h after a similar period of apnea and a decrease in PaO_2_, ~ 23% in BHD, cardiac troponins elevate^9^. This study could not confirm these observations in elite BHD with personal records of static apnea of more than 5 min, and after a similar decrease in PaO_2_, after 4 min of apnea and at end of max apnea. Our observations are also consistent with animal models demonstrating that after the onset of myocardial ischemia, cell death is not immediate but takes a finite period to develop: it has been reported that the level of cardiac troponins I (cTnI) starts to increase by 30 min with a peak value at 3 h after cardiac arrest in adult pigs^30^ and 4 h of hypoxia in rats has no effect on coronary flow rate or cardiac troponin T^31^. cTNI (and Pro-atrial natriuretic peptide) also remain unaffected in competitive BHD 3 h after two consecutive maximum apneas (static apnea ~ 5 min and subsequent ~ 88 m underwater swim) (Kjeld T. et al. 2014). The question is, why the otherwise highly adapted BHD, possessing extreme tolerance for hypoxia^1–5,7^ including a mitochondrial adaption to hypoxia in their skeletal muscles similar to the northern elephant seal^7^, should display or suffer from cardiac injury after only 4 min of apnea^9,10^? However, high altitude permanent residents, chronically exposed to hypoxia, suffer more often from acute coronary syndromes than comparable low altitude populations, probably due to hyperlipidemia^32^ and hence arteriosclerotic coronary disease, and it may be, that the BHD studied by Kyhl et al. and by Eichhorn et al., were not as cardiac healthy as the subjects with normal lipids^33^ and low resting MBF^34^ in our study (Tables 1 and 4) or as well adapted as discussed below^12^.

Diving seals are exposed to repetitive cycles of ischemia and following reperfusion. Similarly sleeping seals experience sleep apnea, but do not increase production of ROS nor suffer systemic or local oxidative damage^15^ in contrast to terrestrial animals with sleep apnea. Joulia et al. demonstrated in BHD that ischemia-modified albumin (IMA), a marker of the release of ROS during hypoxemia, increases less in the BHD who endured apnea for more than 4 min, than compared to those who endure less than 4 min of apnea^12^. Our study included only BHD who endured apnea for more than 5 min, and our results indicated that they have a similar resistance to cardiac hypoxia as observed in adult diving mammals^8^. Our results included increased myocardial perfusion and are in contrast to the studies by Eichhorn et al. and Kyhl et al. of BHD enduring apneas of 4 min or less and reporting signs of myocardial injury and reduced myocardial perfusion^9,10^. However, studies of restrained and sedated seals demonstrate severely reduced myocardial perfusion during static apnea^35^, but sedation depresses the cardiovascular system including the myocardial regional oxygen supply^36^, and cannot be compared to the foraging animal with an increased metabolic demand during their dives. Compared to the lung tissue and skeletal muscles, seal hearts have the lowest levels of Hypoxia-Inducible Factor-1alpha, an adaptive protein to protect against hypoxia^37,38^. This indicates, that the hearts of seals, also sensitive to localized ischemia^39^, do not have decreased myocardial perfusion during their dives, as it would cause myocardial damage. Elite BHD performing maximum apnea, also go through a struggling phase, with muscular tensions, hence also an increased metabolic demand, which in part may explain the increased myocardial perfusion at end of apnea in our study.

Fatty acids are the major energy source for healthy myocardium, but lactate and ketone bodies, can be the energy substrate under certain circumstances^40^. Gormsen et al. demonstrated that ketone bodies increased myocardial blood flow 75% without affecting myocardial fatty acid transport or oxidative capacity in the myocardium of healthy subjects^41^. A novel study demonstrated that ketone bodies can even increase CO and LVEF in patients with heart failure without impairing myocardial external energy efficiency^42^. These observations are in line with our results of elite BHD, demonstrating that after a warm-up and during a consecutive maximum apnea, myocardial perfusion increases, concomitant with a decrease in pH and also a decrease in lactate. A decrease in lactate may be the consequence of decreased production, a higher metabolic rate or both, whereas hypercapnia-induced respiratory acidosis during exercise promote a decrease in blood pH^43^, but a respiratory acidosis have negligible effects on the lactate response^44^. We interpretate the concomitant decrease in pH as a consequence of the increase in PaCO_2_^45^ during decreasing lactate in this study, the latter may indicate lactate metabolization, as discussed below. Similarly, the hearts of harbor seals have a heightened lactate dehydrogenase activity indicating an adaptation for the anaerobic production of adenosine triphosphate (ATP) on dives beyond the animal's aerobic dive limit^8^. Lactate is cumulated during aerobic exercise, and can be metabolized after exercise by skeletal muscle, heart, liver, kidneys, brain, adipose tissue and lungs^46^. However, Kaijser et al. demonstrated that lactate is the primary substrate for oxidation of the heart muscle cells during short-term normoxic heavy exercise in normal young subjects at sea level^47^, and similarly the subjects in our study performed maximum apnea with increases in MBF comparable to the increases in MBF in endurance trained athletes during maximum exercise^29^. Sherpas living in high altitude with chronic hypoxic exposure have also been demonstrated to decrease lactate levels during maximum exercise suggestive of lactate metabolization^48,49^. Hence, we suggest, that the elite BHD in our study have an adaption to metabolize lactate during hypoxia similar to these high-altitude Sherpas and the harbour seal cardiac mitochondria^8^.

Mitochondria are central to synthesis of ATP and ROS, and hence also important to myocyte death^50,51^. Exposing the myocardium to acute ischemia and reperfusion causes initially mitochondrial dysfunction and subsequent excessive production of ROS by the electron transport chain^50^. The deleterious effects of ROS include damage to the mitochondria^52^, resulting in depolarization and apoptosis^53^. These processes may ultimately lead to a compromised cardiac function: Chronic ischemic hearts in animal models also possess a decreased capacity for oxidative phosphorylation^54–57^, whereas hypoxia in a hypobaric environment may trigger increased cardiac carbohydrate metabolism and enhanced mitochondrial respiratory capacity, even with preserved contractile function^58^. Stride et al. demonstrated that chronic ischemic human hearts possess a decreased intrinsic mitochondrial respiration^59^, which ultimately may lead to failure of the left ventricle^60^. In this study of elite BHD during hypoxia, no signs of myocardial ischemia could be demonstrated by CMR or myocardial perfusion deficits by ^15^O-H_2_O-PET/CT, despite a decline in ejection fraction, and hence declined myocardial contractile function concomitant with bradycardia at end of apnea. We suggest that the resistance to even more extreme cardiac hypoxia in our study, than found in previous similar studies^9,10^, at least in part confirms our hypothesis, of elite BHD possessing a cardiac adaptive resistance to hypoxia.

Swine LV volume in a CMR closed-chest model have been demonstrated to decrease ~ 53% during 25 min of untreated ventricular fibrillation, while the mean interventricular width or LV wall thickness markedly increases^61^. At the end of apnea in our study, LVEDV decreased by ~ 49%, concomitantly with an ~ 31% increase in LV wall thickness and a ~ 49% decrease in LVCO which is consistent with the findings in similar studies of BHD^9,10^. Our findings are also comparable to observations in diving adult harbor seals in which thermodilution measured stroke volumes at surface are almost twice the volumes compared to when submerged^62^.

In conclusion, our study demonstrated for the first time cardiac hypoxic resistance and increased MBF in the hearts of elite BHD, during apnea of more than 5 min concomitant with systemic hypoxia below 4.3 kPa ± 0.4 and decreasing lactate. Our results indicate an increased ability for anaerobic and fat-based metabolism in the hearts of elite BHD during hypoxia similar to the harbor seal during routine diving. In elite BHD, biventricular EF and SV decreases at end of maximum apnea concomitant with bradycardia, similar to CMR of hypoxic diving mammals during apnea. Hence, the results of our study confirm, that the hearts of elite BHD have similar adaptions to hypoxia as air breathing diving mammals.

## Perspectives

Future studies may reveal myocardial oxygen extraction at end of apnea in elite BHD, and may confirm presumed hyperdynamic LVEF due to lactate metabolization after apnea in elite BHD.

Furthermore, it is speculated that understanding of these mechanisms may help in the development of new treatments of severe clinical conditions caused by extreme hypoxia, e.g. cardiac surgery, organ transplantation and in the post-resuscitation care setting.

### Limitations

The number of subjects was low because the inclusion criteria required the BHD to have very long apnea, and this may give problems with statistical power. A correlation analysis would be of interest to find a possible graded difference in diving response between subjects, but this would need to have a larger number of subjects with larger differences in levels of apnea. However, the findings were for most analyses statistically significant because of the pronounced physiological effects and each subject being its own control. BOLD on the other hand was not different during apnea compared to baseline and thus this finding should be viewed with some caution due to few subjects. The length of the apneas were different for MR, ^15^O-H_2_O-PET/CT and blood gases in the pool: BOLD-MR and blood gases were both collected at 4 min of breath-hold to be able to compare the results. ^15^O-H_2_O/PET-CT for absolute perfusion cannot be done at one time point but is collected over several minutes. Therefore, we collected data from 2 to more than 8 min of breath-hold. Subjects insisted on drinking coffee on the day for apneas before ^15^O-H_2_O-PET/CT, and therefore MBF may be impaired compared to fasting from caffein^63,64^. In one subject, resting and recovery ^15^O-H_2_O-PET/CT scans were lost due to technical difficulties.

Apnea during exercise with facial immersion have been demonstrated to increase the cardiovascular responses compared to apnea and exercise without facial immersion^65^. However, data collected in our study were both dry and wet static apnea, and a difference may be observed in the cardiovascular responses, if all data could be collected during wet apnea^65^.

## Data Availability

Data collected in this study are all saved encrypted at hospital servers.
