# Author Correction: Cardiac hypoxic resistance and decreasing lactate during maximum apnea in elite breath hold divers

**DOI:** 10.1038/s41598-021-85418-9

**Published:** 2021-03-11

**Authors:** Thomas Kjeld, Jakob Møller, Kristian Fogh, Egon Godthaab Hansen, Henrik Christian Arendrup, Anders Brenøe Isbrand, Bo Zerahn, Jens Højberg, Ellen Ostenfeld, Henrik Thomsen, Lars Christian Gormsen, Marcus Carlsson

**Affiliations:** 1 Department of Radiology, Herlev Hospital, University of Copenhagen, Herlev Ringvej 75, 2730 Herlev, Denmark; 2 Department of AnesthesiologyHerlev Hospital, University of Copenhagen, Copenhagen, Denmark; 3 Department of Cardiothoracic Surgery, Rigshospitalet, University of Copenhagen, Copenhagen, Denmark; 4 Department of Clinical Physiology and Nuclear Medicine, Herlev Hospital, University of Copenhagen, Copenhagen, Denmark; 5 Department of Cardiothoracic Anesthesiology, Rigshospitalet, University of Copenhagen, Copenhagen, Denmark; 6 Department of Clinical Sciences Lund, Skåne University Hospital, Lund University, Lund, Sweden; 7 Department of Nuclear Medicine & PET Centre, Aarhus University Hospital, Aarhus, Denmark

Correction to: *Scientific Reports* https://doi.org/10.1038/s41598-021-81797-1, published online 28 January 2021

This Article contains errors in the values reported in Table 2. In addition, the row ‘Neck saturation / %’ should be omitted.

The correct Table 2 appears below.

**Table 2:** Hemodynamic parameters and blood gas results at rest, after 4 min apnea and at end of apnea (326 ± 19 s).

	Rest	4 min apnea	Max apnea	After apnea
MAP/mmHg	103 ± 4	152 ± 8***	148 ± 15***	119 ± 6
HR/beats min^−1^	86 ± 5	48 ± 3***	50 ± 13***	64 ± 9*
SaO_2_/%	96.7 ± 0.5	76.7 ± 3.4*	51.7 ± 12.9***	N/A
pH	7.42 ± 0.03	7.39 ± 0.02	7.35 ± 0.01***	N/A
PaCO_2_/kPa	5.2 ± 0.2	5.7 ± 0.4***	7.0 ± 0.2***	N/A
PaO_2_/kPa	12.2 ± 0.5	6.5 ± 0.3***	4.3 ± 0.4***	N/A
SBE	0.26 ± 0.60	0.58 ± 0.74***	2.3 ± 0.59***	N/A
Bicarbonate	24.7 ± 0.44	24.4 ± 0.52	24.8 ± 0.51	N/A
Lactate	1.70 ± 0.13	1.33 ± 0.18*	1.36 ± 0.18*	N/A

Values are means ± Standard Error of Means; *P < 0.05 versus rest and ***P < 0.001.

*MAP* mean arterial pressure; *HR* heart rate; *SBE* standard base excess; *SaO*_*2*_ arterial oxygen saturation; *PaCO*_*2*_ partial pressure of carbondioxide; *PaO*_*2*_ partial pressure of oxygen.

